# Prevalence of dual sensory impairment in veterans: a rapid systematic review

**DOI:** 10.3389/fresc.2024.1281491

**Published:** 2024-03-01

**Authors:** Zara Raza, Syeda F. Hussain, Renata S. M. Gomes

**Affiliations:** ^1^ BRAVO VICTOR, Research & Innovation, London, United Kingdom; ^2^ Hull York Medical School, University of York, York, United Kingdom; ^3^ Northern Hub for Veterans and Military Families Research, Department of Nursing, Midwifery and Health, Faculty of Health and Life Sciences, Northumbria University, Newcastle upon Tyne, United Kingdom

**Keywords:** Dual sensory impairment, sensory loss, veterans, military, traumatic brain injury

## Abstract

Dual sensory impairment (DSI) is prevalent in the older population, but due to exposure to military-related risk factors, it is a particular problem for veterans, older and younger. This rapid review aimed to critically review and summarise the prevalence of DSI in military veteran populations, as well as any associative factors and outcomes that were assessed. This was done in accordance with the Preferred Reporting Items for Systematic Reviews (PRISMA) statement. Several databases (Scopus, Web of Science, AMED, CINAHL Plus, Ultimate, and MEDLINE via EBSCOHost) were searched and five studies were selected for final review. All studies provided a prevalence rate for DSI in a veteran sample. One study also looked at functional independence as an outcome. Three of the studies considered blast injuries and traumatic brain injury (TBI) by using samples from TBI patient populations. Overall, results of this review suggest that age and presence of TBI and/or exposure to blast may increase prevalence of DSI in veterans. Prevalence rates ranged from 5.0–34.6% but there are caveats. There is a lack of universal or standardised definition for DSI, making it difficult to determine true prevalence. Future research should also include veterans who may not be receiving support from Veterans Affairs, consider factors such as TBI aetiology and severity based on clinical measures, and utilise a more standardised definition for DSI based on clinical measures.

## 1 Introduction

Concurrent hearing loss and vision loss is referred to as dual sensory impairment (DSI) or dual sensory loss (DSL). In some cases, DSI is also used interchangeably with “multi-sensory impairment” (MSI) although this label lacks a clear universal definition and at times it refers to DSI in addition to other impairments or disabilities (1). Henceforth, in this review we will use the term “dual sensory impairment”. According to SENSE, more than 400,000 individuals in the United Kingdom (UK) experience both these sensory losses and this number is expected to increase to 600,000 by 2030, as a result of an ageing population (2). Individuals with DSI are referred to as deafblind and in 1995, the Department of Health noted that one is regarded deafblind “if their combined sight and hearing impairment cause difficulties with communication, access to information and mobility.” This definition includes those with sight and hearing loss that is progressive and partial.

The type of DSI is based on the onset of the DSI. DSI may be congenital, acquired, or age-related (3). Congenital DSI refers to people who were born with vision impairment (V.I.) and hearing impairment (H.I.) or developed it in the early years before language development. Acquired DSI describes individuals who developed both sensory impairments after language development. Syndromes such as CHARGE syndrome and USHER syndrome may lead to congenital and acquired DSI respectively (3). In this review, all types of DSI were considered. Due to the nature of the population included in this review i.e. the medical requirements for eligibility to join the military, it is not as likely that there will be participants with congenital DSI in comparison to acquired DSI.

There is a significant impact on an individual's life when they develop sight loss or hearing loss. It is therefore worth determining the proportion of the population that are already living with DSI, and the proportion of individuals who are at risk of acquiring DSI as it presents unique challenges, in comparison to those living with single sensory loss. In the general population, the number of older-aged people is increasing (4) and the age profile of the veteran population is changing accordingly. According to Ministry of Defence projections for the UK veteran population, although a drop in veterans age 75 and over is predicted (from 49% in 2016 to 37% in 2028), the percentage of veterans that are 90 years and over is expected to double from 6% in 2016 to 12% in 2028 (5). As sight loss and hearing loss can occur in military personnel during deployment, or training, there is value to examining DSI in the military veteran population. The effects of DSI may interact with the effects of other conditions that occur in military populations, such as traumatic brain injury (TBI). Furthermore, V.I. and H.I. are known outcomes of TBI and exposure to explosives, putting veterans, and military personnel at particular risk of developing DSI unrelated to ageing. With outcomes such as depression (6, 7), dementia (8) and increased risk of mortality (9) being associated with DSI, determining prevalence, and assessing rehabilitation needs that holistically consider such outcomes, especially in a group with additional occupation-related risk factors is important.

Prevalence rates of vision and hearing impairments on their own are difficult to ascertain, and the prevalence of DSI is often underestimated according to a 2018 report by the World Federation of the Deafblind (10). Furthermore, much of the DSI literature is focused on DSI prevalence in the aging population, and in relation to congenital DSI rather than acquired DSI (11). Documentation of DSI in medical records is also lacking (12). Furthermore, self-reporting issues can lead to under or overestimation of DSI. In the military and veteran population, often it is the rates of either V.I. or H.I. or both separately that are recorded even if there are cases of DSI.

## 2 Methods

A rapid systematic review was conducted, two reviewers (ZR and SH) contributed to the title and abstract, quality appraisal and full text-reviews.

### 2.1 Literature search

**Figure 1:**
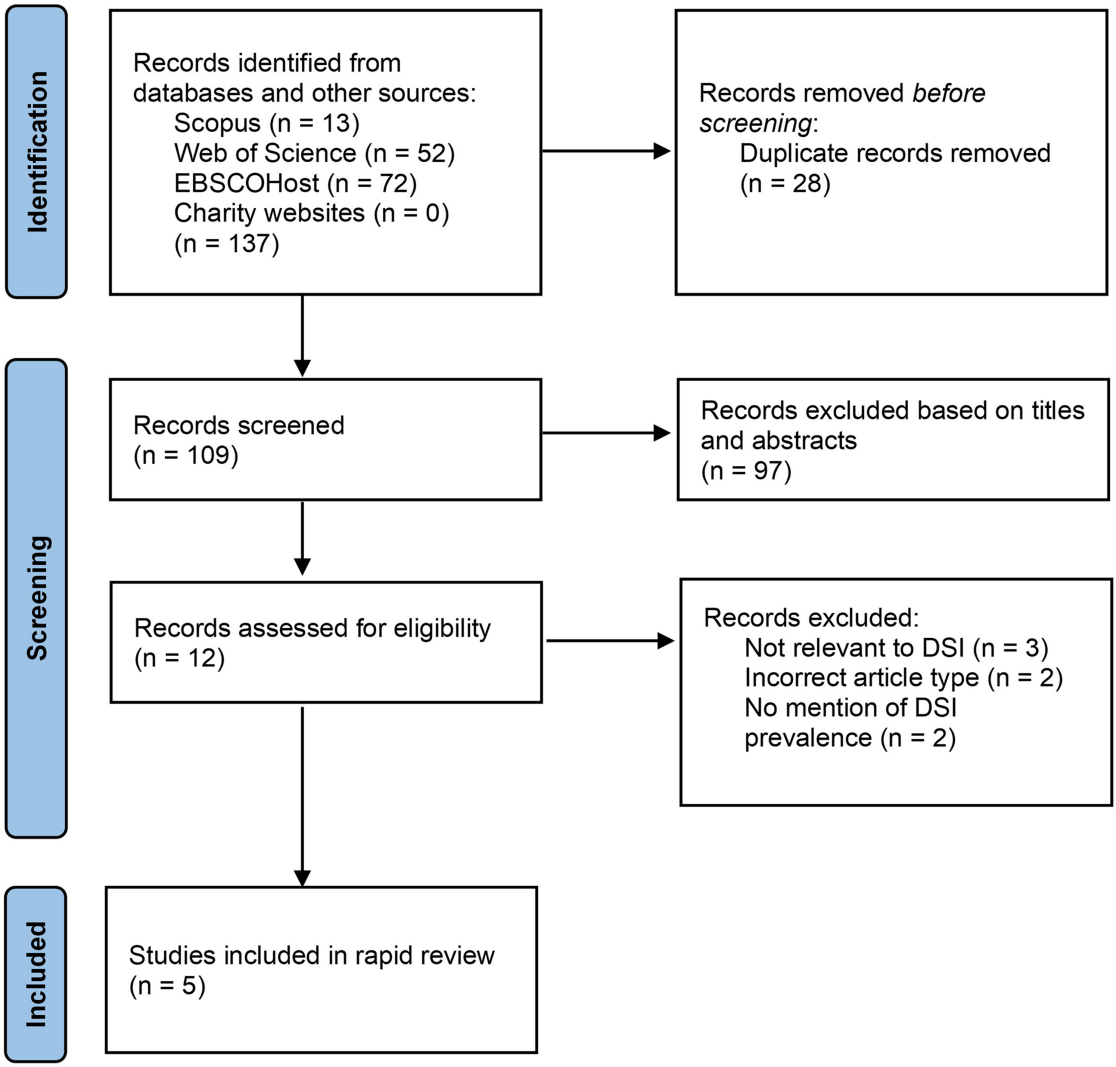
Flow chart diagram based on PRISMA.

The Preferred Reporting Items for Systematic Reviews (PRISMA) (13) was used to conduct this rapid review (Figure 1). A systematic search of literature was performed in different academic databases: Scopus, Web of Science, and AMED, CINAHL Plus and Ultimate, and MEDLINE via EBSCOHost. The following search string was used and adjusted for, per database search requirements:

(dual sensory loss OR dual sensory impairment OR vision and hearing loss OR vision and hearing impairment OR multi-sensory impairment OR multisensory impairment OR deafblind*) AND (veteran* OR ex-military OR ex-forces OR army OR marine* OR soldier* OR air force OR navy OR national guard OR deployment OR combat OR active component OR armed forces)

The search was refined to English language and restricted to articles or reviews only. Duplicate articles were removed.

A search for grey literature was also conducted. The websites of several charities supporting veterans with disabilities were searched for reviews and reports presenting statistics of veterans with DSI.

### 2.2 Inclusion criteria

Studies were included if they: (i) included veterans aged 18 years and older; (ii) presented prevalence statistics of combined vision and hearing impairment, (iii) were written in English. Full-text articles that were peer-reviewed were considered (see Supplementary for reasons for exclusion). Review-type articles were scanned for any relevant citations of studies that presented prevalence statistics.

The titles and abstracts of the articles obtained from the literature search results (*n* = 109) were screened. At this stage, studies that did not refer to vision and hearing loss/impairment, military and/or veterans were excluded. The remaining articles (*n* = 12) were independently reviewed by both reviewers to determine suitability. Articles for which full-article access was not available were excluded. After reviewing the full-text articles, those that were not relevant to DSI, did not contain prevalence statistics for DSI, and were not the correct type of article were excluded. Articles which contained prevalence estimates of DSI in veteran populations were included (*n *= 5).

### 2.3 Data extraction and critical appraisal

Quality appraisal and risk of bias assessment of the studies was carried out by SH and ZR using the appraisal tool for Cross-Sectional Studies (AXIS tool). The studies were appraised based on aim, methods, results, discussion, ethics and funding. Elements of the studies including study design, quality of reporting and potential for bias were gauged to assess the quality of the study. The tool consisted of 20 questions, each having three possible responses (“yes”, “no” and “don't know”), with the score increasing by 1 for each “yes”. Each study was given a score between 0 and 20. The studies were graded as “Good” (>15), “Fair” (10–15) or “Poor” (<10). The following fields were extracted: authors, year of publication, setting/country, study design/type, sample size, participant characteristics (age and sex), and prevalence data.

## 3 Results

Figure 1 shows the flowchart for the studies included and excluded. Excluded articles (12, 14–19) and the reasons for exclusion can be found in the Supplementary. Table 1 outlines the key details about the five studies.

**Table 1:** Study characteristics (participant recruitment, study sample size, country); population characteristics (age range/mean age, sex, grouping); prevalence data and measurement methods for DSI in the selected studies.

Source	Study characteristics	Sample population characteristics	Prevalence (%)	Measurement of DSI	Methodology: strengths and weaknesses
Lucas and Zelaya (20)	• Used data that was collected via the 2016 National Health interview Survey. • *N* = 33,208. • Country: United States.	• Age: 18–75 + years. • Male veterans and non- veterans.	• All percentages have a 95% confidence interval. • 5.0% (3.4–6.9) DSI in veterans. • 2.5% (2.2–2.9) in non-veterans. 18–44 years: 0.6% (0.4–0.9) • in non-veterans. 45–64 years: 5.1% (3.4–7.3) • in veterans and 3.2% (2.6–3.8) in non-veterans. 65–74 years: 6.8% (5.0–8.9) • in veterans and 5.1% (3.9–6.7) in non-veterans. • 75 years and over: 10.6% (8.3–13.2) in veterans and 9.7% (6.8–13.3) in non-veterans.	• Self-reporting questionnaire. Questions asked to evaluate vision and hearing were: “Without the use of hearing aids or other listening devices, is your hearing excellent, good, a little trouble hearing, moderate trouble, a lot of trouble, or are you deaf?”, “Do you have any trouble seeing, even when wearing glasses or contact lenses?”, and “Are you blind or unable to see at all?”. • Categories for degree of hearing were obtained by combining “excellent” and “good” into on category, “a little trouble” and “moderate trouble” into another category, and “a lot of trouble” and “deaf” into another. • Participants who responded to the question about blindness were excluded as blindness could not be differentiated from other degrees of visual troubles, and only 142 respondents selected yes for this question, so could not be analysed separately. • DSI refers to combined V.I. and H.I. of any degree or loss.	Strengths: • Used national sample in the United States. • Large sample size. Weaknesses: • Focused on self- reporting, susceptible to bias. • Relied on subjective categorization of answer options.
Lew et al. (21)	• Used data from TBI evaluations carried out by Veterans Health Administration between October 2007 and June 2009. • *N* = 21,627. • Country: United States.	• Age: 18–65 years (*M* = 31.3, SD = 8.6). • 93.9% male. • Subjects split into two groups: 12,521 patients with deployment- related TBI and 9,106 participants without TBI.	• 35.% DSI in 10,431 patients with TBI and blast exposure. • 30.3% DSI in 2,090 patients with TBI but no blast exposure. • 24.6% DSI in 6,478 patients with blast exposure but no TBI. • 22.7% DSI in 2,628 participants with no TBI and no blast exposure.	• Self-reporting of V.I. and H.I. using five-point Likert-type scale of rating “vision problems, blurring, trouble seeing, hearing difficulty” on a scale ranging from 0 (none) to 4 (very severe). Data was either treated as a quantitative scale or as a dichotomous categorical variable where those selecting “none” and “mild” were combined into one category.	Strengths: • Used national sample in the United States. • Large sample size. Weaknesses: • Focused on self- reporting, susceptible to bias.
Lew et al. (22)	• Used patient medical records of patients with TBI admitted to Department of Veterans Affairs Polytrauma Rehabilitation Centers (PRC) between December 2004 and March 2008. • *N *= 62. • Country: United States.	• DSI noted in 20 patients (32.3%).	• DSI noted in 20 patients (32.3%).	• Hearing component: Hughson Westlake procedure used to measure pure tone thresholds. Integrity of tympanic membrane and middle ear, as well as air and bone conduction also measured. • Vision component: Feinbloom chart was used to measure visual acuity and the ICD-9 was used to define V.I. • No definition of DSI provided by authors.	Strengths: • Used detailed medical records. • Detailed hearing and vison examinations. Weaknesses: • Small sample size. • No definitions of DSI.
Smith et al. (23)	• Used medical records of 1,472 patients seeking treatment at both optometry and audiology outpatient clinics at VA medical centre between June 2004 and May 2005. • *N *= 400. • Country: United States (Mountain Home, Tennessee).	• Patients divided into 4 age groups: <65 years (*n = *505, range = 44–64), 65–74 years (*n *= 363), 75–84 years (*n* = 485), and >85 years (*n* = 199, range = 85–95 years). • 100 patients were randomly selected from each age group.	• Significant increase in DSI prevalence with increasing age. 0% prevalence in <65 age group and 26% prevalence in >85 age group. • <65 years: 0% (PTA and HFPTA) • 65–74 years: 1% (PTA) and 4% (HFPTA) • 75–84 years: 9% (PTA) and 13% (HFPTA). • >85 years: 22% (PTA) and 26% (HFPTA). • Overall DSI prevalence 7.4% (HFPTA) and 5.0% (PTA).	• Hearing component: Measured using both HFPTA and traditional PTA, resulting in different prevalence figures for each. • Vision component: Snellen chart for distance visual acuity. • DSI definition: visual acuity of worse than 20/40 in the better eye and an unaided moderate hearing impairment or worse in the better ear (>40 dB).	Strengths: • Used detailed medical records. • Random patient selection. Weaknesses: • Small sample size. • Restricted to a specific geographical location in the United States.
Pogoda et al. (24)	• Used patient medical records veterans completing Veterans Affairs comprehensive TBI evaluation (CTBIE) between October 2007 and June 2009. • *N* = 13,746. • Country: United States.	• Age: 18–64 years (male patients had *M* = 31.4, SD = 8.7 years). • Sample split into two groups for comparison: 9,998 patients with mTBI history and 3,748 patients with no history of TBI	• Overall MSI rate of 13.9%. • MSI rate in patients with deployment-related mTBI (both blast and non-blast) was 17.4%.	• Self-reporting of V.I. and H.I. via the use of the Neurobehavioral Symptom Inventory (NSI-22, a 22-item patient self-report checklist). • Participants were asked to rate the degree of trouble in the last 30 days for “hearing difficulty”, “vision problems, blurring, trouble seeing”, and “feeling dizzy”, “loss of balance” and “poor coordination, clumsy” in reference to vestibular issues. These were rated on 5-point Likert scales ranging from 0 (none) to 4 (very severe). • Symptoms that were reported at least a 2 were categorised as moderate impairment. • MSI refers to combined reporting of V.I., H.I. and vestibular symptoms, with a score of 2 or greater for all three symptoms.	Strengths: • Used detailed medical records. • Large national sample Weaknesses: • Focused on self- reporting, susceptible to bias. • Relied on subjective categorization of answer options.

### 3.1 Critical appraisal

The AXIS tool was used to assess the quality and risk of bias. The quality of studies ranged from Fair to Good. Four studies (22–24) received a score of 10–15 (Fair) and one (23) received a score >15 (Good). In the Good study, some of the following strengths were identified: objective measurement of exposure and outcome, appropriate statistical analyses, and identification of important confounding factors. The weaknesses noted in these studies however were small sample sizes, bias introduced by subjective measurements, and the lack of universal definition for DSI.

### 3.2 Description of studies

#### 3.2.1 Topic and aim

All five studies' primary aim was to determine estimates of prevalence rates for V.I., H.I. and DSI in veteran populations. Three of the studies (21, 22, 24) investigated DSI specifically in veteran populations that had received TBI evaluations. One (22) considered the effects of TBI-related sensory impairments on functional independence. All studies identified for reviewing in the present article are (descriptive) cross-sectional studies.

#### 3.2.2 Participants

Participants in three studies (21, 22, 24) all had TBI evaluations, whereas the other two studies looked at general veteran populations. However, all but one study (20) involved data obtained from veterans who were seeking healthcare from the Department of Veterans Affairs (VA). Lucas & Zelaya used data obtained via a health survey administered on a national level i.e., not limited to veterans that are under the care of VA healthcare. Most of the studies used a relatively large sample size ranging from 13,746 to 36,919, but two (22, 23) used much smaller samples. Smith et al. looked at 400 participants, with group sizes of only 100 participants per group. This is due to stratifying their population according to age, with one of the age-groups acting as a limiting factor. Lew et al. (2009) however looked at only 62 patients, and no justification for this was provided. All studies included participants that were 18 years and older, except for Smith where the youngest participants were 44 years, but the eldest were 95 years old. Two studies had similar average ages as well as similar age ranges for participants [18–65, *M* = 31.3, SD = 8.6 and 18–64, 31.4, SD = 8.7 for Lew et al. (2011) and Pogoda respectively].

#### 3.2.3 Research design

A descriptive cross-sectional design was used for all articles. This design is ideal as they are prevalence studies, and the databases used generally allow for substantial sample sizes. All studies used patient data from databases, except Lucas & Zelaya who used survey data.

### 3.3 Methods

#### 3.3.1 Measuring and defining visual impairment

In Smith et al.'*s* study, distance visual acuity was evaluated using a Snellen chart, whereas a Feinbloom chart at 10 meters was used to assess distance visual acuity in Lew et al. (2009)'s study (visual acuity was based on best corrected refraction of the better eye and dependent on existing corrections). Near acuities were also obtained by Lew et al. (2009), and these were measured using text, single words, triple digits, or single digits. Where visual field results were available, Smith et al. relied on the Humphrey Automated Visual Field Analyzer or the manual Goldmann perimeter to review visual field results contributing to V.I. Lew et al. (2009) do not refer to visual field, and only mention the use of visual acuity as a measure of visual function. The remaining three studies measured V.I. using self-reporting criteria such as the validated NSI-22 inventory (24), or referring to reports of “vision problems, blurring, trouble seeing” over a period of the past 30 days (21), or answering questions such as “Do you have trouble seeing, even when wearing glasses or contact lenses?” (20).

The International Classification of Diseases—9th revision (ICD-9) was used to define V.I. by Lew et al. (2009) as it was reflective of the eligibility criteria of the VA for accessing vision rehabilitation. Visual acuity of 20/20 to 20/63 was defined as normal and near normal vision. Visual acuities between <20/63 and 20/1,000 were categorised into a single group consisting of those with moderate, severe and profound V.I. based on ICD-9 definitions. Finally, a visual acuity of <20/1,000 (or bilateral enucleation) indicated blindness. These latter two groups would be eligible to access VA vision rehabilitation services. Smith et al. defined V.I. as “best corrected visual acuity of worse than 20/40 in the better eye” and included the US definition of legal blindness (best corrected visual acuity of ≤20/200 in the better eye or visual field of less than 20°). Similar to Lew et al. (2009) they also categorised patients into three categories based on visual acuities, i.e., normal and near-normal (≥20/40), vision impairment (20/50 to 20/100) and legal blindness (≤20/200). Both definitions classify V.I. based on the better eye. No such categorisation was available for the other three articles as no standardised definition was used for these.

#### 3.3.2 Measuring and defining hearing impairment

The audiometric tests referred to by Lew et al. (2009) and Smith et al. were carried out using Grason-Stadler GS1 Model 61 audiometers in a double-walled, sound-treated booth. They both used the modified Hughson Westlake procedure to measure pure tone thresholds. They differed in their other measurements however. Smith et al. assessed word recognition using the Northwestern University Auditory Test No. 6, Lew et al. (2009) do not mention assessment of this. Lew et al. (2009) did however discuss measurement of peripheral hearing function via air- and bone-conduction thresholds assessment, and measurement of the integrity of the tympanic membrane and middle ear using the QT1 Quik Tymp Tympanometer.

Lew et al. (2009) labelled different levels of H.I. severity based on the lowest hearing threshold (measured in dB) in the poorer ear at any frequency (frequency thresholds used were 250, 500, 1,000, 2,000, 4,000 and 8,000 Hz). The severities were mild hearing loss (HL) (26–40 dB), moderate HL (41–60 dB), severe HL (61–90 dB) and profound HL (>90 dB). In contrast, Smith et al. based their definition of H.I. on the better ear to remain consistent with the definition for V.I. H.I. was defined using the unaided pure tone average (PTA) of thresholds at 500, 1,000, and 2,000 Hz (frequencies represented in speech). The severities were defined as normal (<25 dB), mild (25–40 dB), moderate (41–55 dB), moderate-severe (56–70 dB), severe (71–90 dB) and profound (>90 dB). The authors however investigated another PTA at higher frequencies (high-frequency PTA or HFPTA) i.e., the thresholds this time included 1,000, 2,000, and 4,000 Hz (frequencies associated with speech in background noise). HFPTA was assessed as an alternative metric because PTA is considered to be a less valid indicator for H.I. i.e., a significant proportion of participants that relied on hearing aids were being classified as being in the normal hearing range when using PTA.

Similar to the V.I. assessments, the remaining three studies relied again on self-reporting in order to determine presence of H.I.

#### 3.3.3 Defining DSI

A working definition of DSI was established by Smith et al. for the study, defined as visual acuity of worse than 20/40 in the better eye and an unaided moderate hearing impairment or worse (>40 dB hearing level PTA) in the better ear. Lew et al. (2009) did not discuss a working definition of DSI for their paper and it is assumed they included those who had both V.I. and H.I. according to their criteria for each to have DSI. There was no clarification of which severities of V.I. and H.I. would lead to an individual being classified as having DSI. Furthermore, in the remaining three studies, participants were identified as having DSI if they reported to have both V.I. and H.I. according to the respective self-reporting criteria.

### 3.4 Prevalence of DSI

Prevalence of DSI in Lew et al. (2009)'s population of veterans with blast-related TBI was found to be 32.3% (*n* = 20). The authors made no comparisons to veterans with no TBI. The prevalence of DSI amongst those with deployment-related TBI (both blast exposed and non-blast exposed) in Lew et al. (2011) sample was found to be 35% (22.7% for those with no TBI and no blast exposure, 30.3% for those with TBI but no blast exposure, and 35.4% for those with TBI and blast exposure). Smith et al. found a significant increase in DSI prevalence with increasing age. There was a 0% prevalence in those aged under 65 and a 26% prevalence in those aged over 85 using high frequency pure tone average (HFPTA). Overall DSI prevalence was 7.4% using HFPTA or 5% using traditional PTA when measuring H.I. All individuals aged over 65 in the study who had vision impairment or legal blindness were also noted to have a moderate or worse hearing impairment based on HFPTA. Therefore, in this age group, the prevalence of V.I. and DSI was the same. Male veterans in Lucas and Zelaya's study had a DSI prevalence rate of 5.0%. The prevalence rate provided by Pogoda is for MSI. It was 13.9% for the general sample, and 17.4% in the mild traumatic brain injury (mTBI) subgroup (participants having deployment-related mTBI and both non-blast and blast injuries). This estimate however cannot be used interchangeably with DSI prevalence rates since this set of sensory impairments includes cases of participants vestibular impairment in addition to V.I. and H.I. Despite the fact that MSI is perhaps a more comprehensive term, these rates are not comparable to the DSI rates found in the other studies since they are measuring different things.

### 3.5 Predictors of DSI

Lew et al. (2011) carried out separate multiple linear regressions to investigate the following predictor variables for sensory impairments: age, gender, impairment of the other sensory modality, blast exposure, TBI and two- and three-way interactions among TBI status, blast exposure and gender. The key finding was that V.I. and H.I. were significantly correlated [*r* (21,625) = .33, *p* < .0001], meaning that having H.I. was a predictor for having V.I., and vice versa. The suggested possible reasoning for this was that the source for both impairments may be the same or that both systems may be impaired by the same cause. Lucas and Zelaya compared prevalence of DSI according to age and veteran status and found that male veterans were more likely to have DSI than non-veterans (5.0% vs. 2.5%), but similar prevalence of DSI were found in veterans and non-veterans when stratifying by age. Pogoda carried out logistic regression regression via three models to evaluate the predictive effect of certain factors. They concluded that significant predictors of reporting MSI were older age (those 40 years and older were nearly thrice as likely to report MSI than 18–24 year olds), being female (45% more likely than males to report MSI after accounting for all other factors), lower military rank, injury aetiology, deployment-related mTBI history, PTSD, and depression. The most robust significant predictor was found to be mTBI history when considered alongside PTSD and depression.

#### 3.5.1 TBI severity

As TBI is a known cause of DSI (14), three of the five studies considered if not the severity, but at least the presence of TBI in their populations. Lew et al. (2009) categorised TBI severity as mild, moderate or severe using the Glasgow Coma Scale—a widely accepted diagnostic scale for assessing TBI severity, duration of posttraumatic amnesia (PTA), and duration of posttraumatic loss of consciousness (LOC). In cases where all three diagnostic criteria were not recorded, the most severe categorisation of the three criteria was used to inform the severity. Lew et al. (2011) TBI deployment-related classification was based more on self-reporting of exposure to blast and clinical judgement of TBI, and severity was not accounted for. Pogoda further restricted those with deployment-related TBI to those who met the criteria for mild TBI (mTBI) based on Veterans Affairs Department of Defense clinical practice guidelines that are in concordance with American Congress of Rehabilitation Medicine criteria. There was also no categorisation according to severity in this study. All three studies identified TBI as a predictor for DSI or MSI.

#### 3.5.2 TBI and DSI

H.I. and dysfunction following TBI is common whether the injury is blast- or non-blast related, mild or severe (25, 26). This can occur due to fracturing of the temporal bones or injury to the auditory nerve, and the hearing loss is usually sensorineural (26–28). Vision impairment following a TBI is also a common outcome and may occur via damage to the ocular structure or cortical components of the visual pathway (29). The two types of sensory impairment therefore can overlap and co-occur in civilians and veterans who have sustained a blast- and non-blast related TBI. Indeed, three of the five studies included TBI context for their study samples. Lew et al*.* (2009) was the most comprehensive in that they attempted to categorise the severity level of TBI according to three well-known diagnostic criteria. They observed equal proportions of patients with DSI in the mild and severe TBI groups (36% per group) but only 17% of those with a moderate TBI had DSI. However, there were just over twice as many patients with mild and severe TBIs in comparison to moderate TBI.

In Lew's 2011 paper, authors calculated prevalence data in relation to presence or absence of self-reported blast exposure, and presence or absence of TBI. This was helpful in further elucidating how likely DSI is to occur as a function of two known risk factors. Their data revealed the highest prevalence of DSI for those with TBI and blast exposure, and the lowest for those with no TBI and no blast exposure. The prevalence was always the greatest when TBI was present. Pogoda similarly considered injury aetiology (blast, non-blast or both) along with TBI. Their results also showed a higher rate of MSI in those that had a history of mild TBI and a history of both blast and non-blast exposure. Whilst Lew et al. (2011) saw a greater prevalence of DSI in TBI-diagnosed patients with blast exposure in comparison to no blast exposure, Pogoda did not find any evidence for MSI being more likely for one aetiology over the other. Furthermore, a key finding of Lew et al. (2011) was that the greatest variance was found for H.I. rather than V.I. when taking into account blast exposure. Furthermore, it is important to reiterate that DSI as defined by Lew et al. is different to MSI as defined by Pogoda.

Further research would benefit from exploring the impact of both TBI severity and aetiology. Additionally, considering the likelihood of veterans with TBI having both visual and auditory impairments, there should be a systematic screening and periodic evaluations of vision and hearing following exposure to incidences that may have caused a TBI such as a blast, physical assault, motor vehicle accident etc. Lew et al. (2009) found a 22.7% DSI prevalence rate in those without TBI and no blast exposure, and this points to the possibility of other military-related causes (e.g., protective equipment, environment, training) of H.I. and V.I. that also need to be taken into account.

#### 3.5.3 Age and DSI

Dual sensory loss can present at any age but its prevalence has been found to increase significantly from the youngest to the oldest age groups in civilian populations (30–32). This trend would undoubtedly be reflected in the veteran population, however, there may be differences in the age of onset of DSI and causes of DSI. In Smith et al.'s study their veteran population was stratified according to the following age groups: < 65 years (44–64), 65–74, 75–84, and 85 + years (85–95). Authors calculated a prevalence rate of 22%–26% in the >85-years group, 9%–13% for 75 to 84 years, 1%–4% for 65 to 74 years, and 0% in the <65-years strata. Lew et al. report that the ages of their 62 patients ranged from 19 to 47 years, and the mean age was 27.3 years (SD = 7.0 years). Lew et al.'s study seems to indicate loss of vision and hearing being prevalent in the younger demographic of veterans whereas the other study points to DSI being prominently prevalent in older veterans. However, the age range was smaller for Lew et al. (2009)'s study in comparison to the Lew et al. (2011), Smith and Pogoda where the average age range was about 50 years. The mean age was also relatively young at 27.3 years (SD = 7.0 years). Lucas and Zelaya did not provide an age range or a mean age, but categorised into four groups: 18–44, 45–64, 65–74 and 75 + . Comparisons based on age between these cannot be explicitly made because of the lack of standardised categorisation of age, as well as the way in which risk factors such as TBI were accounted for in only some studies. Future research should utilise a larger age range (18 to >80 years) that would allow for comparison between various military cohorts in different countries. The identification and assessment of all confounding factors is also vital as these can impact comparability and extrapolation to wider populations.

### 3.6 Outcomes of DSI

**Figure 2:**
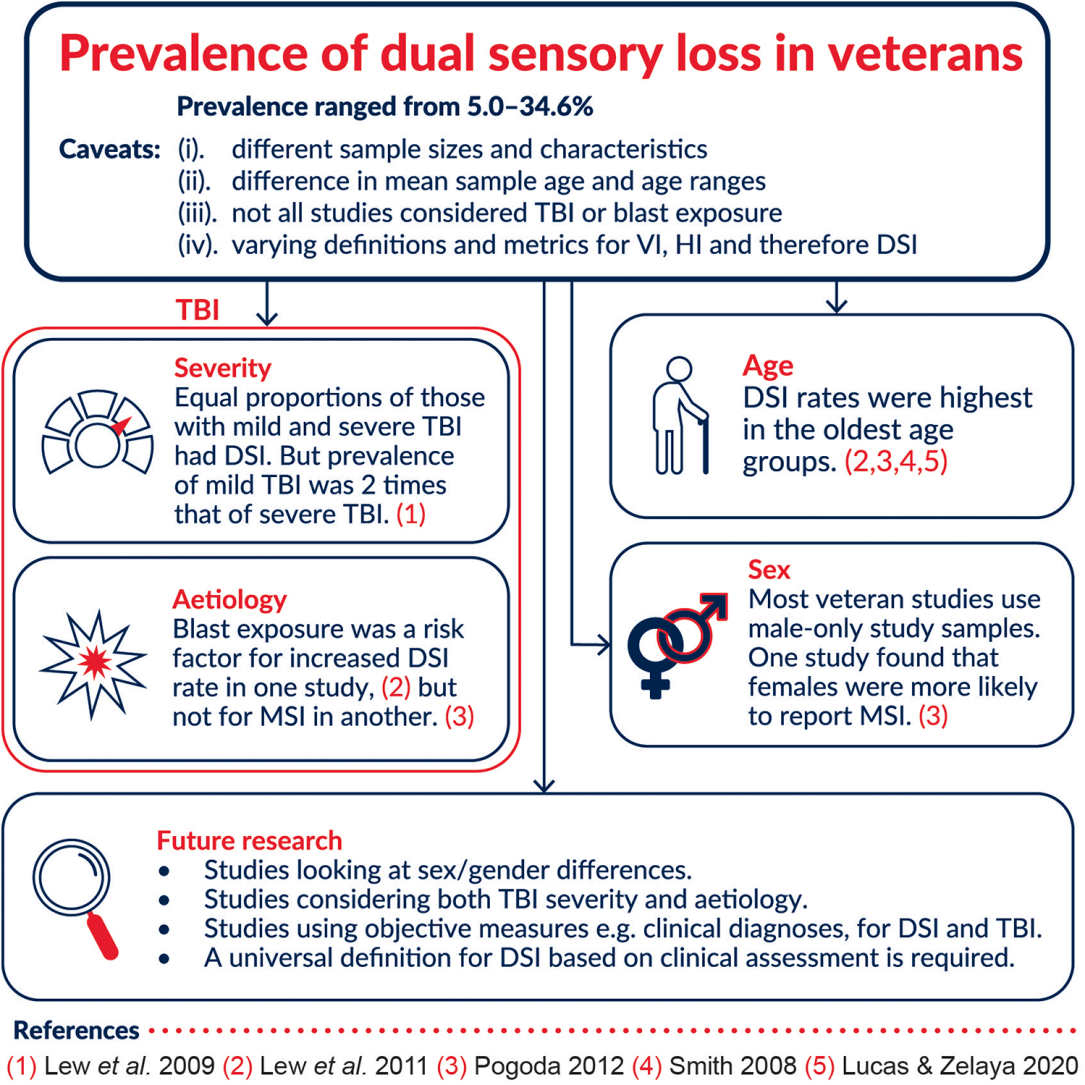
Summary of prevalence and related factors identified in the studies reviewed.

Of the five studies, only Lew et al. (2009) investigated any outcomes of DSI in the veteran population. The authors looked at functional independent measures (FIM) which consisted of total, motor and cognitive scores. ANOVA and Tamhane's T2 post hoc *T*-tests were used to determine the relationship between sensory impairment types and changes in FIM. FIM was measured at admission and discharge. Change in FIM between admission and discharge was also measured. Generally, at admission, there were no statistically significant differences in FIM scores between the groups (no sensory impairment, V.I. only, H.I. only and DSI). At discharge, the total and motor FIM were marginally lower in those with DSI than those with no sensory impairment (NSI) (total DSI = 107.7 ± 19.2, total NSI = 120.1 ± 6.0, motor DSI = 79.7 ± 15.4, motor NSI = 89.0 ± 4.6, *p* < 0.09). DSI patients had lower scores for all subgroups within motor FIM (self-care, mobility, locomotion, and sphincter control). However, the group mean values were only significantly different in the self-care subgroup [*F* (3, 58) = 2.93, *p* < 0.05]. Although not statistically significant, the DSI group did show slightly lower scores for the cognitive components at discharge. DSI was also a significant contributor to decreased total and motor FIM score changes after completing regression analysis but did not have a significant effect on cognitive change (Figure 2).

## 4 Discussion

### 4.1 Prevalence rates of DSI

Four of the studies provided a prevalence rate for DSI in their respective populations, whilst one reported for MSI which is defined as having at least two forms of sensory impairment (vestibular, visual, and auditory).

The patients in the sample for Smith, Lew et al. (2009), Lew et al. (2011) and Pogoda's studies were all receiving VA support. These are evidently not general veteran populations as they're solely inclusive of veterans who are seeking VA healthcare. In comparison to veterans who are not receiving VA healthcare, these veterans may have access to evaluations and interventions that non-VA healthcare receiving veterans do not have access to. This could lead to an underestimation in the prevalence of DSI or an underestimation of the effects of DSI. In comparison to civilian studies where the cause of DSI is most likely to be age-related, in veteran populations, especially those who are receiving VA healthcare, causes such as trauma and head injuries may also be a prevalent cause. A large-scale European study found an overall prevalence rate for self-reported DSI of 7.54% (95% CI: 7.36–7.72) in adults, and 14.78% (95% CI: 14.35–15.21) in the 70 or over age group. This and many other studies of its kind demonstrate that DSI prevalence increases with age. Similarly, in most of the studies looked at in this review, age was considered as a control when investigating the effects of other factors. In veterans however, TBI is an important factor to consider as military personnel are a known at-risk group for head injuries, hence the VA usually screen for TBI upon admission. Lucas and Zelaya's was the only study that made comparisons to non-veterans, and veteran status was confirmed only via the national health questionnaire i.e., the veterans in this sample may or may not have been receiving healthcare from the VA so is perhaps more representative of the general US veteran cohort. Aside from Lew et al. (2009)'s study sample, all the other studies used a large sample size, although methods are not comparable since Smith, for example, limited group sizes according to the lowest number of participants in each age group. Lew et al. (2009)'s prevalence rate is therefore not generalisable to a TBI veteran population. The authors also suggest a possible underestimation of H.I. due to central auditory testing not being carried out, which in turn could result in an underestimation of DSI.

The prevalence calculated from these studies cannot be compared on the basis that each study considered varying risk factors for DSI, made different group comparisons, measured different variables, measured variables using different methods, and sample frames were different in terms of size and the population bases from which they were obtained.

### 4.2 Inconsistency in defining DSI

Many studies that assess for DSI and its outcomes often rely on self-reporting as opposed to measuring or assessing vision and hearing. Lew et al. (2009) did not clarify their definition of DSI but did outline the assessment methods used by the clinics to define V.I. and H.I. severities. They also outline a need for establishing an operational definition for H.I. and DSI. Smith et al. highlight that there is no standardised method of defining and measuring DSI, and even use two different methods for measuring the H.I. component of DSI to demonstrate how prevalence estimations can be affected by the complexity defining DSI. According to their study, the HFPTA is a more valid indicate for H.I. than PTA. Whilst TBI was assessed through clinical judgement, V.I. and H.I. status was based on self-reports in Lew et al.'s 2011 study. Lucas and Zelaya, and Pogoda also relied on self-reporting of visual and hearing symptoms to determine V.I. and H.I. status. With the dependence on self-reporting, it is possible to introduce bias by the way of social desirability bias or lack of introspective ability. Furthermore, it is impossible to confirm self-reported symptoms. In Pogoda et al. the definition of DSI differed to that used by the rest of the studies assessed for this review. Systematic reviews (32, 33) that looked at DSI prevalence also emphasised the lack of comparability between studies due to the vast differences in DSI metrics. This undoubtedly contributes to the paucity of prevalence data for DSI. Without a universal definition of DSI, it is also difficult to implement any changes that could benefit those that are affected by DSI. Therefore, it is necessary for the scientific and clinical communities through a task group initiative, to agree on international diagnostic standards and specific methods for future application.

### 4.3 Study quality and risk of bias

Lew et al. (2011) had the lowest score (14 out of 20), followed by Lew et al. (2009), Lucas and Zelaya, and Pogoda (15/20), and Smith's study was the highest with a score of 16. When conducting the critical appraisal, one of the main critiques of the studies by Lucas and Zelaya, Pogoda and Lew et al. (2011) was their inclusion of self-reported outcomes for V.I. and H.I. These were subjective measurements instead of objective measurements that reduce the reliability of the articles and introduce self-reporting bias. V.I. and H.I. were established by trusting the patient's own disclosure when asked survey-style questions. Therefore, for future research, it would be better to avoid using questionnaires when assessing these parameters. One of the other common critiques identified for most of the studies was that the sample frame was taken from VA healthcare-receiving veterans, meaning that results may not be extrapolated to the general veteran population in the US. The only study to not extract participants from this particular population was the one by Lucas and Zelaya, as this study utilised a nationwide health survey. However, for this study, veteran status was assessed subjectively, and only male subjects were included.

### 4.4 Grey literature

Websites of several charities that support ex-service personnel, and veterans living with disabilities (for example Forces in Mind Trust, Help for Heroes, BLESMA, Royal British Legion) were searched for reviews and reports presenting statistics of veterans with DSI. However, none were found. Of the literature that did refer to prevalence of sensory issues (34–38), they only focused on instances of V.I. or H.I. independently, rather than the numbers of individuals with DSI. It would be beneficial for charities and organisations that support veterans to keep records of DSI.

### 4.5 Limitations and future research

Although this study is a rapid review, one of the limitations is that only few databases were used to conduct the searches. The use of a greater number of databases may have resulted in a greater number of studies that fit the inclusion criteria. The searches were also limited to English language, and this may have led to relevant international studies in different languages, focusing on different populations being missed.

Studies have been conducted with a number of civilian populations (39) to determine relationships between DSI and quality of life, however as we have identified from conducting this review, this has not been extended to the veteran population. There is a need for understanding these relationships in this population considering the military-related risk factors (exposure to physically and mentally adverse events), comorbidities (for example post-traumatic stress disorder) and disabilities (e.g., limb loss) that can compound the effects of DSI to significantly impact quality of life.

Moreover, given that magnetic resonance imaging (MRI) is widely used for assessments of TBI, and TBI is in turn a predictor for DSI it would be valuable to include DSI measures in TBI studies. Furthermore, acquiring ocular and inner ear MRI images during MRI TBI investigations would be important for future research, especially for understanding of cause, causation, and correlation between TBI and DSI.

## 5 Conclusion

This review highlights a lack of reliable data on the prevalence of DSI in veterans, perhaps unsurprising considering a similar trend in non-veteran populations. Based on the five studies used in this review, DSI prevalence in veterans is estimated to be 5.0%–34.6% but with some caveats: (1) sample sizes and characteristics were considerably different, (2) difference in mean sample age and age ranges make it difficult to compare, especially with age being a risk factor for DSI (3) only some studies used TBI patient groups and considered blast exposure, and these factors may contribute to higher DSI prevalence rates in some studies (4) the definitions and metrics for V.I., H.I. and therefore DSI differed, with one study even comparing two different methods of measuring H.I. thus resulting in two different prevalence rates (Figure 2). There are numerous studies that report on the prevalence of DSI in civilians in various countries and regions. However, aside from the US to the best of our knowledge, there are no similar studies in veteran populations elsewhere. Ageing is an ever-present risk factor for DSI, but in the military population it is important to note that there is a high risk of being exposed to events that are likely to lead to sensory loss such as training, blasts and explosions. While a few studies did explore the predictive factors for DSI in veteran populations, there was variability in the range of factors explored and in the measurements of these factors. This warrants further investigation on the true prevalence of DSI in military and veteran populations as well as the effects on quality of life, and rehabilitation needs of veterans.

## Data Availability

The original contributions presented in the study are included in the article/Supplementary Material, further inquiries can be directed to the corresponding author.

## References

[1] Kamenopoulou L Ali A Ockelford A. Multi–sensory impairment: convenient label or recipe for confusion? A scoping review of research conducted in England (2001–20). J Res Spec Educ Needs. (2021) 21:98–110. 10.1111/1471-3802.12503

[2] https://www.sense.org.uk/information-and-advice/conditions/deafblindness/.

[3] Janssen MJ Hartshorne TS Wittich W. Editorial: development, wellbeing, and lifelong learning in individuals with a dual sensory loss. Front Psychol. (2021) 12:790549. 10.3389/fpsyg.2021.790549 34956018 PMC8695599

[4] Office for National Statistics. Voices of our ageing population: Living longer lives. (2022). Available online at: https://www.ons.gov.uk/peoplepopulationandcommunity/birthsdeathsandmarriages/ageing/articles/voicesofourageingpopulation/livinglongerlives (accessed January 23, 2024).

[5] Ministry of Defence. Population Projections: UK Armed Forces Veterans residing in Great Britain, 2016 to 2028. (2019) Available online at: https://assets.publishing.service.gov.uk/government/uploads/system/uploads/attachment_data/file/775151/20190107_Enclosure_1_Population_Projections_-_UK_Armed_Forces_Veterans_residing_in_Great_Britain_-_2016_to_2028.pdf (accessed January 23, 2024).

[6] Capella-McDonnall ME. The effects of single and dual sensory loss on symptoms of depression in the elderly. Int J Geriatr Psychiatry. (2005) 20(9):855–61. 10.1002/gps.1368 16116571

[7] Guthrie DM Declercq A Finne-Soveri H Fries BE Hirdes JP. The health and well-being of older adults with dual sensory impairment (DSI) in four countries. PLoS One. (2016) 11(5):e0155073. 10.1371/journal.pone.0155073 27148963 PMC4858206

[8] Kuo PL Huang AR Ehrlich JR Kasper J Lin FR McKee MM Prevalence of concurrent functional vision and hearing impairment and association with dementia in community-dwelling medicare beneficiaries. JAMA Netw Open. (2021) 4(3):e211558. 10.1001/jamanetworkopen.2021.1558 33739429 PMC8601132

[9] Zhang X Wang Y Wang W Hu W Shang X Liao H Association between dual sensory impairment and risk of mortality: a cohort study from the UK biobank. BMC Geriatr. (2022) 22:631. 10.1186/s12877-022-03322-x 35915397 PMC9341066

[10] World Federation of the Deafblind. At risk of exclusion from CRPD and SDGs implementation: Inequality and Persons with Deafblindness. Initial global report on situation and rights of persons with deafblindness September (2018) Available online at: https://www.wfdb.eu/wp-content/uploads/2019/06/WFDB_complete_Final.pdf (accessed January 23, 2024).

[11] Tiwana R Benbow SM Kingston P. Late life acquired dual-sensory impairment: a systematic review of its impact on everyday competence. Br J Vis Impair. (2016) 34(3):203–13. 10.1177/0264619616648727

[12] Dullard B Saunders GH. Documentation of dual sensory impairment in electronic medical records. Gerontologist. (2016) 56:313–7. 10.1093/geront/gnu032 24846883 PMC7289325

[13] Moher D Liberati A Tetzlaff J Altman DG Group P. Preferred reporting items for systematic reviews and meta-analyses: the PRISMA statement. Br Med J. (2009) 339:b2535. 10.1136/bmj.b2535 19622551 PMC2714657

[14] Lew HL Weihing J Myers PJ Pogoda TK Goodrich GL. Dual sensory impairment (DSI) in traumatic brain injury (TBI)–an emerging interdisciplinary challenge. NeuroRehabilitation. (2010) 26(3):213–22. 10.3233/NRE-2010-0557 20448311

[15] Halbauer JD Ashford JW Zeitzer JM Adamson MM Lew HL Yesavage JA. Neuropsychiatric diagnosis and management of chronic sequelae of war-related mild to moderate traumatic brain injury. J Rehabil Res Dev. (2009) 46(6):757–96. 10.1682/JRRD.2008.08.0119 20104402

[16] Saunders GH Echt KV. Blast exposure and dual sensory impairment: an evidence review and integrated rehabilitation approach. J Rehabil Res Dev. (2012) 49(7):1043–58. 10.1682/JRRD.2010.08.0157 23341278

[17] Swan AA Nelson JT Pogoda TK Amuan ME Akin FW Pugh MJ. Sensory dysfunction and traumatic brain injury severity among deployed post-9/11 veterans: a chronic effects of neurotrauma consortium study. Brain Inj. (2018) 32(10):1197–207. 10.1080/02699052.2018.1495340 30024786

[18] Aggarwal V Sashindran VK Dudeja P. Health-care needs and morbidity profile of the elderly veterans and their dependents staying in an urban area:a cross-sectional study. J Mar Med Soc. (2020) 22(1):40–3. 10.4103/jmms.jmms_4_19

[19] Carpenter JG Ersek M Nelson F Kinder D Wachterman M Smith D A national study of end-of-life care among older veterans with hearing and vision loss. J Am Geriatr Soc. (2020) 68(4):817–25. 10.1111/jgs.16298 31886557 PMC13261735

[20] Lucas JW Zelaya CE. Hearing difficulty, vision trouble, and balance problems among male veterans and nonveterans. Natl Health Stat Report. (2020) 142:1–8. PMID: 32600517

[21] Lew HL Pogoda TK Baker E Stolzmann KL Meterko M Cifu DX Prevalence of dual sensory impairment and its association with traumatic brain injury and blast exposure in OEF/OIF veterans. J Head Trauma Rehabil. (2011) 26:489–96. 10.1097/HTR.0b013e318204e54b 21386715

[22] Lew HL Garvert DW Pogoda TK Hsu PT Devine JM White DK Auditory and visual impairments in patients with blast-related traumatic brain injury: effect of dual sensory impairment on functional independence measure. J Rehabil Res Dev. (2009) 46:819–26. 10.1682/JRRD.2008.09.0129 20104405

[23] Smith SL Bennett LW Wilson RH. Prevalence and characteristics of dual sensory impairment (hearing and vision) in a veteran population. J Rehabil Res Dev. (2008) 45:597–609. 10.1682/JRRD.2007.02.0023 18712645

[24] Pogoda TK Hendricks AM Iverson KM Stolzmann KL Krengel MH Baker E Meterko M Lew HL. Multisensory impairment reported by veterans with and without mild traumatic brain injury history. J Rehabil Res Dev. 2012;49:971–84. 10.1682/JRRD.2011.06.0099 23341273

[25] Oleksiak M Smith BM St Andre JR Caughlan CM Steiner M. Audiological issues and hearing loss among veterans with mild traumatic brain injury. J Rehabil Res Dev. (2012) 49:995–1004. 10.1682/JRRD.2011.01.0001 23341275

[26] Lew HL Jerger JF Guillory SB Henry JA. Auditory dysfunction in traumatic brain injury. J Rehabil Res Dev. (2007) 44:921–8. 10.1682/JRRD.2007.09.0140 18075949

[27] Chen JX Lindeborg M Herman SD Ishai R Knoll RM Remenschneider A Systematic review of hearing loss after traumatic brain injury without associated temporal bone fracture. Am J Otolaryngol. (2018) 39:338–44. 10.1016/j.amjoto.2018.01.018 29506762

[28] Shangkuan WC Lin HC Shih CP Cheng CA Fan HC Chung CH Increased long-term risk of hearing loss in patients with traumatic brain injury: a nationwide population-based study. Laryngoscope. (2017) 127:2627–35. 10.1002/lary.26567 28322446

[29] Hussain SF Raza Z Cash ATG Zampieri T Mazzoli RA Kardon RH Traumatic brain injury and sight loss in military and veteran populations- a review. Mil Med Res. (2021) 8:42. 10.1186/s40779-021-00334-3 34315537 PMC8317328

[30] Dawes P Dickinson C Emsley R Bishop PN Cruickshanks KJ Edmondson-Jones M Vision impairment and dual sensory problems in middle age. Ophthalmic Physiol Opt. (2014) 34:479–88. 10.1111/opo.12138 24888710 PMC4273649

[31] Schneider J Gopinath B McMahon C Teber E Leeder SR Wang JJ Prevalence and 5-year incidence of dual sensory impairment in an older Australian population. Ann Epidemiol. (2012) 22:295–301. 10.1016/j.annepidem.2012.02.004 22382082

[32] Minhas R Jaiswal A Chan S Trevisan J Paramasivam A Spruyt-Rocks R. Prevalence of individuals with deafblindness and age-related dual-sensory loss. J Vis Impair Blind. (2022) 116:36–47. 10.1177/0145482X211072541

[33] Heine C Browning C. Dual sensory loss in older adults: a systematic review. Gerontologist. (2015) 55:913–28. 10.1093/geront/gnv074 26315316

[34] The Royal British Legion. Lost Voices. A Royal British Legion report on hearing problems among Service personnel and veterans. Available online at: https://storage.rblcdn.co.uk/sitefinity/docs/default-source/campaigns-policy-and-research/lost_voices_hearing_loss_report.pdf?sfvrsn=5ef1d43a_0 (accessed January 23, 2024).

[35] The Royal British Legion. A UK Household Survey of the Ex-service Community. (2014). Available online at: https://www.britishlegion.org.uk/get-involved/things-to-do/campaigns-policy-and-research/policy-and-research/the-uk-ex-service-community-a-household-survey (accessed January 23, 2024).

[36] The Royal British Legion. Health, welfare and social needs of the Armed Forces community: a qualitative study. Available online at: https://www.britishlegion.org.uk/docs/default-source/campaigns-policy-and-research/welfare_2010_qualitative_study.pdf?sfvrsn=b533fa83_2 (accessed January 23, 2024).

[37] The Royal British Legion. Profile of the Ex-Service Community in the UK. Available online at: http://web1-rbl.temporarywebsiteaddress.com/docs/default-source/campaigns-policy-and-research/profile-of-the-ex-service-community-in-the-uk.pdf?sfvrsn=db2e873_2

[38] Aloni R Levin Y Uziel O Solomon Z. Premature aging among trauma survivors-the longitudinal implications of sleep disruptions on telomere length and cognitive performance. J Gerontol B Psychol Sci Soc Sci. (2021) 76(2):262–72. 10.1093/geronb/gbz077 31155651 PMC8046532

[39] Tseng YC Liu SH Lou MF Huang GS. Quality of life in older adults with sensory impairments: a systematic review. Qual Life Res. (2018) 27(8):1957–71. 10.1007/s11136-018-1799-2 29404924

