# Supplementary material for: Prevalence of dual sensory impairment in veterans: a rapid systematic review
Source: Front Rehabil Sci. 2024 Mar 1;5:1281491. doi: 10.3389/fresc.2024.1281491 (PMC10940421; doi:10.3389/fresc.2024.1281491)
Supplement: Supplementary file 1 [file Table1.docx]

Supplementary Material

**Prevalence of dual sensory impairment in veterans: A rapid systematic review.**

Zara Raza, Syeda F Hussain, Renata SM Gomes^*^

*** Correspondence:** Corresponding Author: Renata.gomes@bravovictor.org

# Supplementary Table 1. Table of excluded articles and reasons for exclusion (n = 7).

| **Excluded Articles** | **Reasons for Exclusion** |
| --- | --- |
| Halbauer 2009 | Not relevant to DSI. |
| Lew 2010 | Not the correct study type; it’s a review type article. Scanned for relevant references to DSI prevalence. |
| Saunders 2012 | Not the correct study type; it’s a review type. Scanned for relevant references to DSI prevalence. |
| Dullard 2016 | No mention of prevalence; its purpose is to determine if there is proper documentation of DSI. |
| Swan 2018 | Not relevant to DSI. |
| Aggarwal 2020 | Not relevant to DSI – only refers to VI and HI prevalence. |
| Carpenter 2020 | No mention of prevalence. Scanned for relevant references to DSI prevalence but none found. Only reference to evaluation of care by family members of individuals with DSI. |
